# MALDI-TOF MS and genomic analysis can make the difference in the clarification of canine brucellosis outbreaks

**DOI:** 10.1038/s41598-020-75960-3

**Published:** 2020-11-06

**Authors:** David Attuy Vey da Silva, Holger Brendebach, Josephine Grützke, Ralf Dieckmann, Rodrigo Martins Soares, Julia Teresa Ribeiro de Lima, Lara Borges Keid, Dirk Hofreuter, Sascha Al Dahouk

**Affiliations:** 1 Department of Biological Safety, German Federal Institute for Risk Assessment, Berlin, Germany; 2 Department of Preventive Veterinary Medicine and Animal Health, Faculty of Veterinary Medicine and Animal Science, University of São Paulo, São Paulo, Brazil; 3 Department of Veterinary Medicine, Faculty of Animal Science and Food Engineering, University of São Paulo, Pirassununga, Brazil; 4 Department of Internal Medicine, RWTH Aachen University Hospital, Aachen, Germany

**Keywords:** Genomic analysis, Mass spectrometry, Sequencing, Microbiology techniques, Bacteria, Bacteriology, Infectious-disease diagnostics, Pathogens, Diagnostic markers, Microbiology, Bacterial infection, Diseases, Infectious diseases, Pathogenesis, Infection

## Abstract

Brucellosis is one of the most common bacterial zoonoses worldwide affecting not only livestock and wildlife but also pets. Canine brucellosis is characterized by reproductive failure in dogs. Human *Brucella canis* infections are rarely reported but probably underestimated due to insufficient diagnostic surveillance. To improve diagnostics, we investigated dogs in a breeding kennel that showed clinical manifestations of brucellosis and revealed positive blood cultures. As an alternative to the time-consuming and hazardous classical identification procedures, a newly developed species-specific intact-cell matrix-assisted laser desorption/ionization–time of flight mass spectrometry analysis was applied, which allowed for rapid identification of *B. canis* and differentiation from closely related *B. suis* biovar 1. High-throughput sequencing and comparative genomics using single nucleotide polymorphism analysis clustered our isolates together with canine and human strains from various Central and South American countries in a distinct sub-lineage. Hence, molecular epidemiology clearly defined the outbreak cluster and demonstrated the endemic situation in South America. Our study illustrates that MALDI-TOF MS analysis using a validated in-house reference database facilitates rapid *B. canis* identification at species level. Additional whole genome sequencing provides more detailed outbreak information and leads to a deeper understanding of the epidemiology of canine brucellosis.

## Introduction

Brucellosis is a widespread zoonosis in livestock, mainly in cattle, sheep as well as goats, and pigs caused by *Brucella abortus, Brucella melitensis*, and *Brucella suis*, respectively. Humans are usually infected through direct animal contact or by the consumption of unpasteurized dairy products and undercooked meat. Both, the lack of surveillance and control of the classical *Brucella* species in some regions^1^ and the lack of awareness of novel and atypical species^2,3^ contribute to an underestimation of the actual prevalence of brucellosis worldwide, especially in developing countries.

Canine brucellosis was first described in the late 1960s^4^ and *Brucella canis* has been identified as the most common cause of brucellosis in dogs with breeding kennels showing high prevalence rates all over the world^5–7^. The infection is transmitted among dogs by intercourse, ingestion of abortion material or reproductive secretions. Moreover, urine of affected dogs is infectious due to contamination with semen^8,9^. The control of canine brucellosis is difficult because dogs maintain close contacts within the population and common management practices in breeding kennels favor the spreading of disease^10^.

Besides *B. canis*, *Brucella suis* has been reported as another cause of canine brucellosis^11–13^. Especially *B. suis* biovar (bv) 1, commonly isolated from feral pigs (*Sus scrofa*)^14,15^, was found in infected dogs^12,16^. In contrast to *B. canis*, which mainly infects domestic dogs, *B. suis* infections occur in hounds that participate in wild boar hunting or are fed with raw boar meat^16–18^. *B. suis* bv 4, the causative agent of reindeer brucellosis in Alaska, Canada and Russia was isolated from wolves, the native relative of dogs, and other carnivores like foxes and wolverines^19,20^.

*Brucella canis* is presumed to be less pathogenic to humans than *B. suis.* However, *B. canis* may be transmitted from dogs to humans, and not only mild clinical symptoms, such as fever, headache, anorexia and fatigue, have been reported in patients, but also severe and/or chronic manifestations, such as peritonitis^21^, endocarditis^22^, osteomyelitis^23^ and arthralgia^24^. Likewise, the virulence of *B. suis* varies between its five biovars, with biovar 1 being highly pathogenic to humans and responsible for severe and chronic courses of the disease^25–27^.

Considering the emerging zoonotic potential of *B. canis* as well as *B. suis* bv 1 and 4, diagnostic tools are necessary to monitor and control canine brucellosis. Unfortunately, infected dogs cannot be easily distinguished from non-infected animals by clinical examination due to the high number of asymptomatic dogs^8,28^. Therefore, fast and reliable laboratory tests are indispensable for the identification of dogs infected with *B. canis* or *B. suis* to contain outbreaks within the dog population and prevent transmission to humans. Since *B. canis* lacks the immunodominant O-polysaccharide of the smooth *Brucella* species^29^, the standard serological tests based on a *B. abortus*-antigen, which are routinely used for the diagnosis of human brucellosis cases, cannot detect anti-*B. canis* antibodies^30^. Hence, the diagnosis of a *B. canis* infection in humans remains a challenge.

The objective of our study was to compare the proficiency of traditional and new omics-based methods in identifying the infectious agent in Brazilian dogs that showed brucellosis-like disease manifestations.

## Results

### Serological examination of dogs with reproductive failure

**Figure 1:**
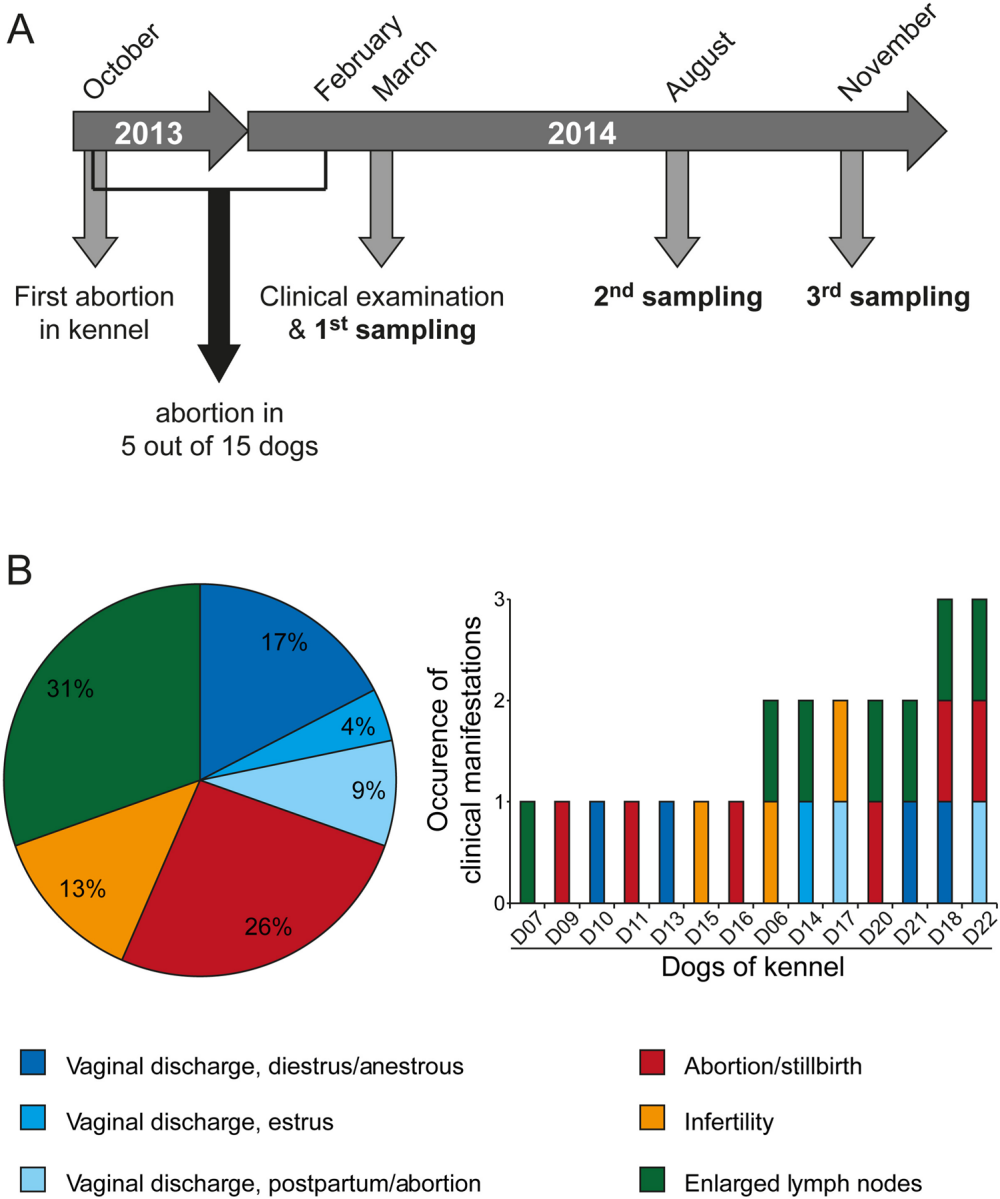
Suspected outbreak of canine brucellosis in a breeding kennel in São Paulo, Brazil. (**A**) Schedule of clinical investigations to clarify the suspected canine brucellosis outbreak. (**B**) Clinical manifestations observed in 14 out of 17 dogs in the kennel.

In 2013, the pregnancy of a female dog in a breeding kennel in São Paulo, Brazil, comprising 17 adult pugs, 2 males and 15 females, resulted in abortion. Another 5 pregnancies ended in abortion in the following four months (Fig. 1A). To clarify whether the observed abortions were a consequence of *B. canis* infections, all animals were clinically examined. While three dogs showed no clinical manifestations related to canine brucellosis, 14 dogs suffered from at least one brucellosis-like symptom (Fig. 1B). Vaginal discharge and general lymphadenopathy were the most common clinical manifestations, followed by abortion/stillbirth and infertility (defined as inability to get pregnant and produce viable offspring) (Fig. 1B).

**Figure 2:**
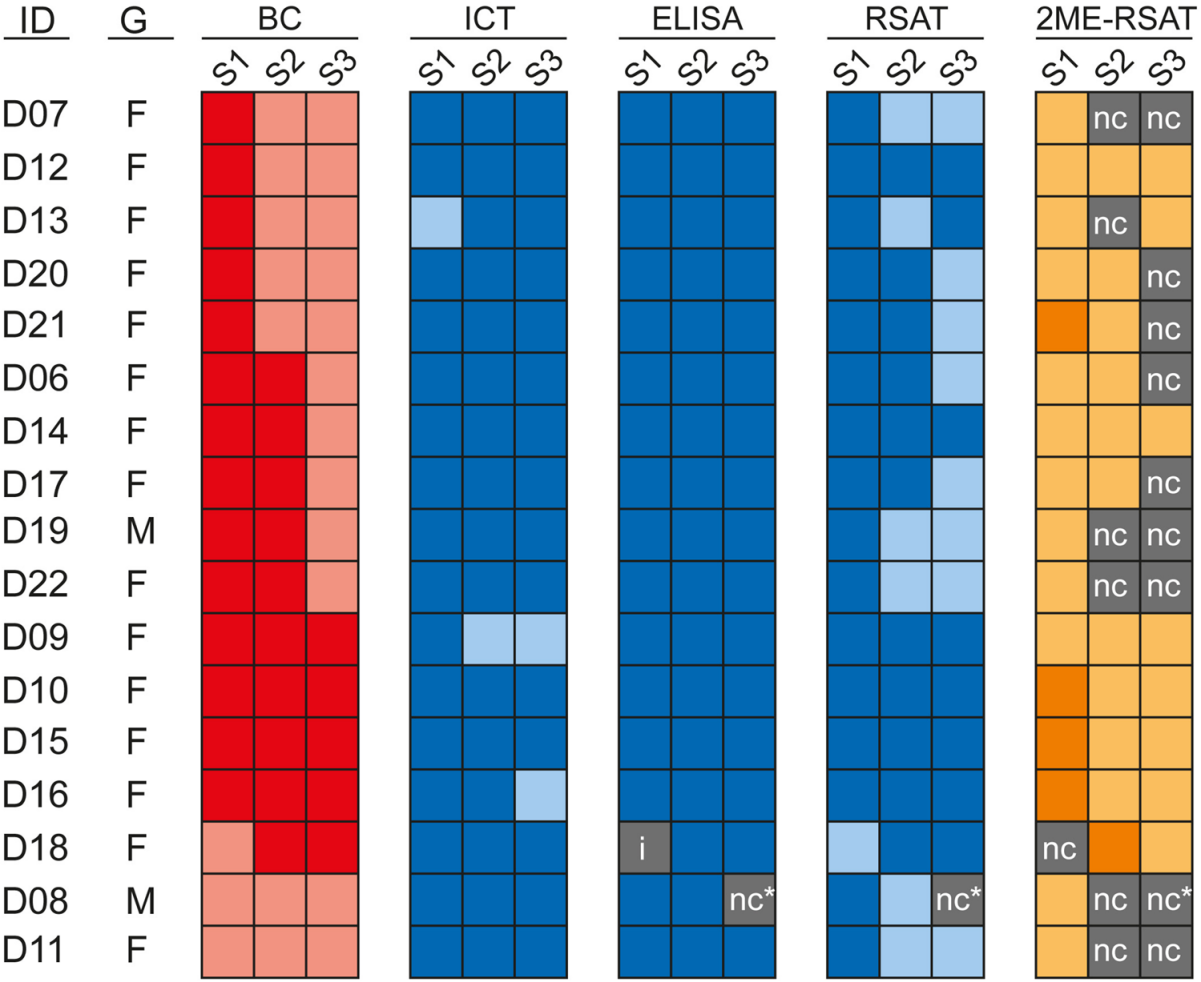
Blood culture and serological test results of dogs from a kennel with a suspected outbreak of canine brucellosis. To determine whether the 17 dogs from the kennel under study were infected with *Brucella*, whole blood samples were incubated in selective medium (BC). Additionally, dogs were serologically tested using an immunochromatographic test (ICT), the rapid slide agglutination test (RSAT), the 2-mercapthoetanol rapid slide agglutination test (2ME-RSAT) and an enzyme-linked immunosorbent assay (ELISA). Positive test results are shown in dark and negative ones in light color. A few tests gave inconclusive results (i) or were not conducted (nc); ID: identification code of the dogs; G: gender (M: male, F: female); S1, S2, S3: sampling dates in March, August and November 2014, respectively; ^*^no serum sample available.

To test whether the animals had developed an immune response against *Brucella* antigens, we collected serum samples from each dog at three consecutive time points (Fig. 1A and Supplementary Table S1) and subjected them to classical serological tests, namely the Immunochromatographic Test (ICT), the Rapid Slide Agglutination Test (RSAT) and a *B. canis* IgG ELISA. In all 17 dogs, we detected antibodies towards rough *Brucella* antigens at each sampling date with at least one of the three conducted serological tests (Fig. 2). In several cases, serological results were equivocal, because the tests revealed contradictory results. For example, the serum from the bacteremic dog D09 did not consistently react against the rough antigen of *Brucella* in the ICT at every sampling date, but in RSAT and ELISA. At the same time, when 2-mercaptoethanol (2ME-RSAT) was applied, which inactivates IgM in the RSAT, we could not detect any antibody reaction in the serum of this dog (Fig. 2).

### Phenotypic characterization of *Brucella* isolates

To assess the infection status of the kennel and to check whether the disease symptoms were indeed a result of an infection with *B. canis*, blood samples were taken from each individual animal at three different time points and aliquots were plated on *Brucella*-selective agar after enrichment broth culture. Gram-negative, short rod-shaped, non-motile bacteria were isolated from 15 out of 17 dogs at least once. A total of 29 isolates from 51 blood samples were obtained (Fig. 2 and Supplementary Table S1) and identified by genus-specific PCR as *Brucella*. Subsequently, genomic DNA was extracted from the 22 blood samples with negative culture results. In three of these blood samples (13.6%) the IS711 marker gene of *Brucella* could be amplified by PCR (Supplementary Fig. S1), which suggests *Brucella* infection in these dogs although the isolation of the bacterium had failed.

**Figure 3:**
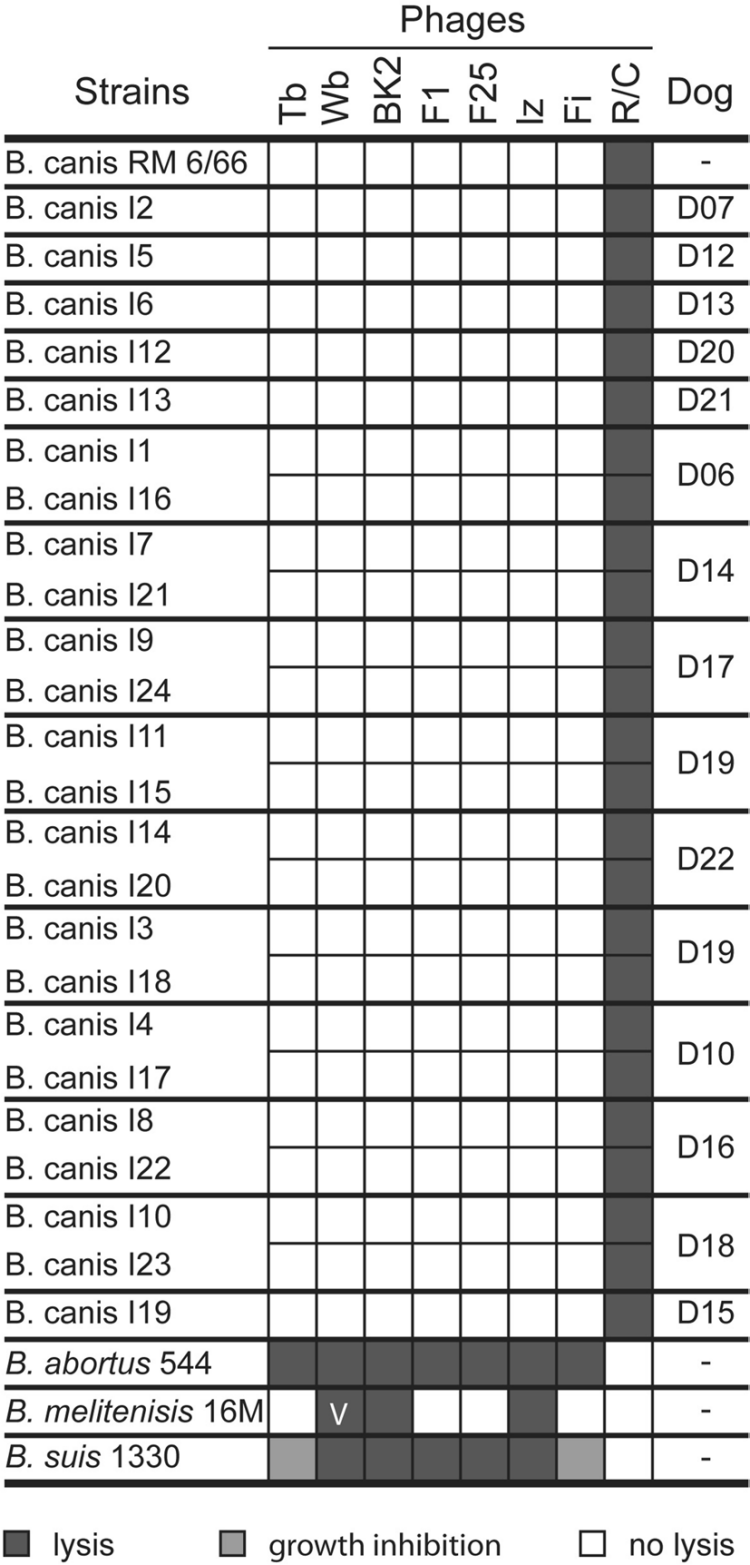
Phage typing of *Brucella* isolates. Presented are the phage lysis patterns of the *B. canis* isolates after incubation with the bacteriophages Tb, Wb, BK2, F1, F25, Iz, Fi, and R/C. Black squares indicate lysis of the bacteria, and white squares indicate no lysis; v: variable. The phage lysis patterns of the reference strains *B. canis* RM6/66, *B. melitensis* 16 M, *B. abortus* 544, and *B. suis* 1330 are shown as determined in our study.

The isolated bacteria were further characterized with phenotyping methods commonly used for the identification of the genus *Brucella* and subtyping of its species and biovars (Supplementary Table S2). The isolates did not require CO_2_ for growth and could be cultured in ambient air (21% O_2_) on blood agar as well as on *Brucella* Agar with or without serum. The bacteria showed oxidase, catalase and urease activity but no H_2_S production similar to the reference strain *B. canis* RM 6/66. Moreover, the isolates grew on thionine and basic fuchsin-supplemented *Brucella* Agar plates comparable to *B. canis* RM 6/66. Additional phage typing experiments revealed the same lysis patterns for the isolated bacteria (constantly lysed by phage R/C, but not by any other tested phage) and *B. canis* RM 6/66 (Fig. 3 and Supplementary Table S3). The isolates did not agglutinate with anti-A and anti-M but with anti-R sera, specific for *Brucella* species expressing rough lipopolysaccharide (LPS). Likewise, LPS phenotyping with crystal violet staining revealed that all isolates expressed a rough LPS, which is characteristic of *B. canis* and *B. ovis*.

Taken together, the classical microbiological methods clearly diagnosed an infection of the dogs with *Brucella* and suggested *B. canis* as the disease-causing agent.

### MALDI-TOF MS-based identification of the canine *Brucella* isolates

**Figure 4:**
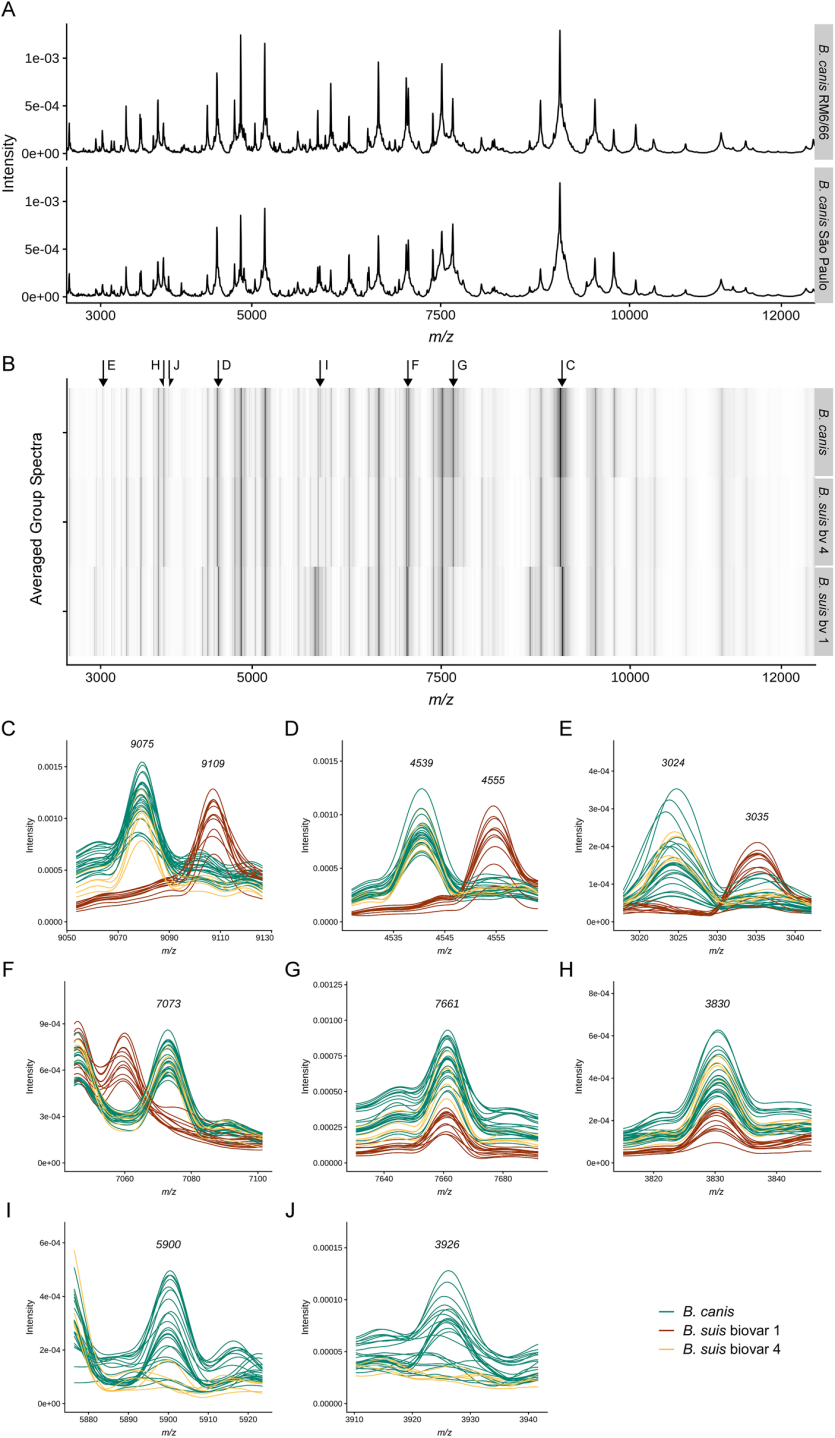
Identification of *Brucella canis* from diseased dogs by MALDI-TOF MS. (**A**) Comparison of pre-processed and normalized spectra of the reference strain *B. canis* RM 6/66 and a representative isolate from the kennel under investigation in the m/z range 3–12 kDa; (**B**) Gel view depiction of averaged group spectra. Arrows indicate m/z positions with high divergence in either intensity or mass-to-charge ratio between *B. canis*, *B. suis* bv 4 and *B. suis* bv 1; (**C**) A biomarker for discrimination of *B. canis* and *B. suis* bv 4 versus *B. suis* bv 1 found by Karger et al.^32^ and its double (**D**) and triple charged (**E**) ions (both from this study); (**F**) The mass peak at m/z 7073 is a unique biomarker for the group *B. canis* and *B. suis* bv 4; (**G**) *B. canis* and *B. suis* bv 4 may be discriminated from *B. suis* bv 1 by their higher intensity mass peaks at m/z 7661 (single charged) and (**H**) m/z 3830 (double charged). (**I** + **J**) Peaks at m/z 5900 and m/z 3926 discriminate *B. canis* from *B. suis* bv 4.

To establish an alternative to the traditional time-consuming *Brucella* diagnostic methods that are based on phenotypic testing, we took advantage of MALDI-TOF MS. Identifying closely related *Brucella* species beyond the genus level with MALDI-TOF MS has been challenging^31^ since only few *Brucella* entries are available in the Security-Relevant reference library used by the Bruker Biotyper software (Bruker Daltonics). Consequently, the here described *Brucella* isolates (Supplementary Table S4) were initially misidentified as *B. melitensis* with scores between 2.0 and 2.3, although their mass spectral patterns were noticeably different from that of *B. melitensis* 16 M (Supplementary Fig. S2) but highly similar to *B. canis* RM 6/66 (Fig. 4A). It has been shown that in particular the discrimination of *B. canis* from *B. suis* bv 3 and bv 4 by MALDI-TOF MS is very difficult^32–34^ even with customized spectral libraries. We therefore improved MALDI-TOF MS-based identification of *Brucella* spp. by optimizing a previously established in-house spectral library^32^ with modified scoring weights for discriminant m/z peaks in the Biotyper software. Our *B. canis* isolates shared a highly similar mass spectral profile with *B. suis* bv 4 strains (Fig. 4B) as described^32^, but could be clearly distinguished from *B. suis* bv 1 (Fig. 4B), which is one of the frequently found *Brucella* species in dogs apart from *B. canis*.

By visual comparison of the MALDI-TOF MS spectra we were able to identify additional discriminant biomarker signals for *B. canis* and *B. suis* besides the previously described biomarkers at m/z 5833 and m/z 9076 specific to *canis-vs-suis* bv 1^32^, corresponding to m/z 5834 (Supplementary Fig. S3A) and m/z 9075 (Fig. 4C), respectively. Both peaks represent single charged molecules and we could also detect their corresponding double or triple charged ions at m/z 2915, m/z 4539 and m/z 3024 (Supplementary Fig. S3B and Fig. 4D,E), respectively. In particular, the peak signals at m/z 9109 (single charged) and m/z 4555 (double charged) occurred prominently in *B. suis* bv 1 but not in *B. canis* or *B. suis* bv 4.

*Brucella canis* and *B. suis* bv 4 share a unique biomarker at m/z 7073 allowing for distinction from *B. suis* bv 1 (Fig. 4F) and other *B. suis* biovars (Supplementary Fig. S4). Further comparison of spectra revealed many differences in peak intensities rather than a strict peak absence or presence. Hence, the peaks at m/z 7661 (single charged) and m/z 3830 (double charged) can be used for the discrimination of *B. canis* and *B. suis* bv 4 against *B. suis* bv 1 (Fig. 4G,H) as well as other *B. suis* biovars (Supplementary Fig. S4). While an accurate and unambiguous distinction between *B. canis* and *B. suis* bv 4 by MALDI-TOF MS has been difficult in previous studies^32^, the isolates used in this study exhibited two signals at m/z 5900 and m/z 3926 that were mainly found in *B. canis* but not in *B. suis* bv 4 strains (Fig. 4I,J).

In summary, our MALDI-TOF MS-based characterization of the *Brucella* isolates from diseased dogs enabled fast and reliable identification beyond the *Brucella* genus level and had sufficient discriminatory power to distinguish *B. canis* from the closely related *B. suis* bv 1 as well as *B. suis* bv 4 isolates.

### Genomic and phylogenetic characterization of the *Brucella canis* isolates

**Figure 5:**
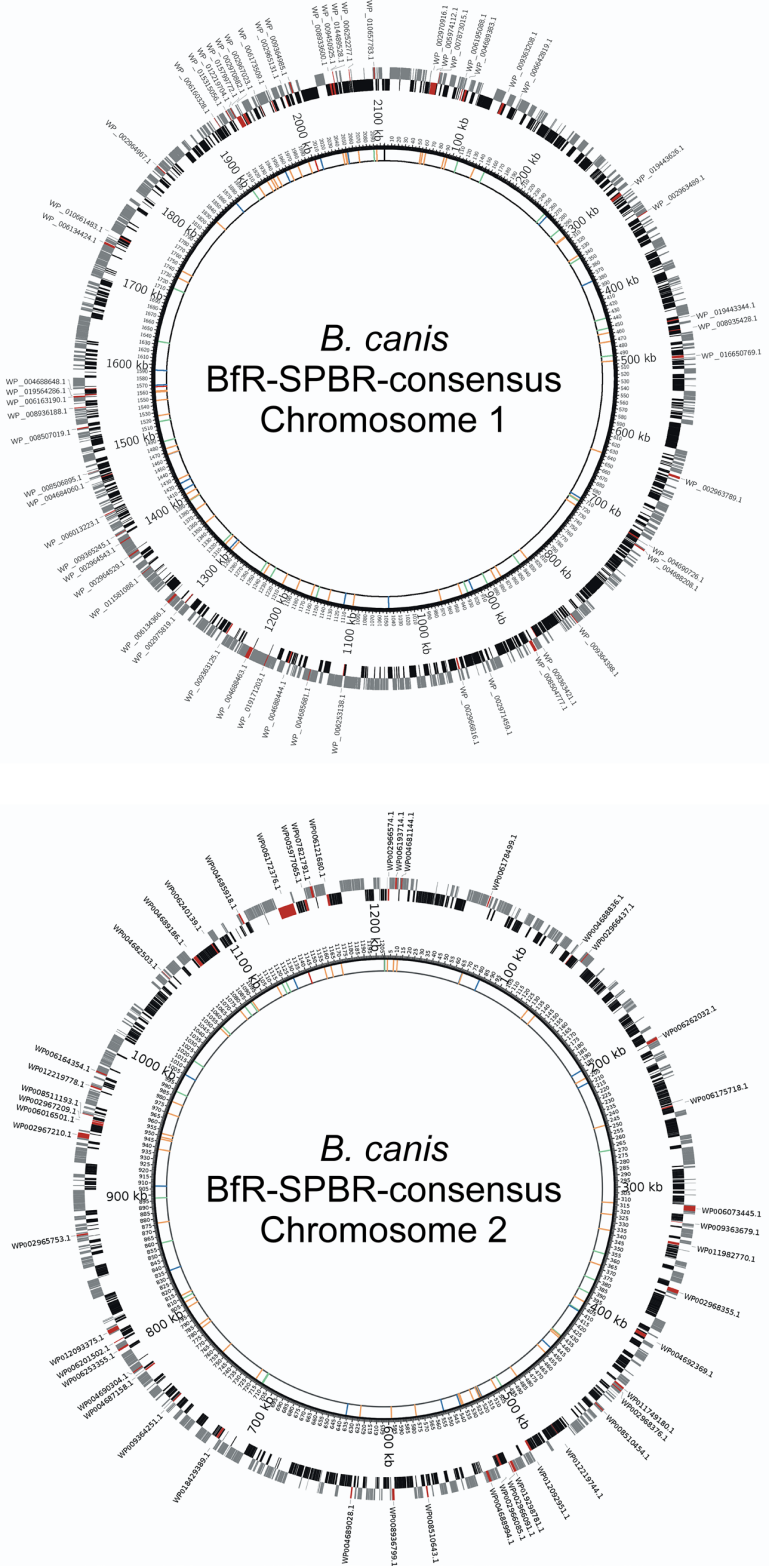
Consensus genome sequence of the *Brucella canis* outbreak strain from a kennel in São Paulo, Brazil**.** Shown are the two chromosomes of the *B. canis* BfR-SPBR-consensus genome derived from whole genome sequencing results of four *B. canis* isolates. The genomes were sequenced using Illumina NGS technology. The open reading frames (CDS) determined by Prokka v1.12^89^ are presented in the two outer circles with grey (+ strand) and black (-strand) boxes. Positions with nucleotide sequence variations in the *B. canis* BfR-SPBR-consensus genome compared to the reference strain *B. canis* ATCC 23365 are depicted in the inner circle and marked with the colors red (stop-loss variants), orange (missense variants), blue (upstream or downstream variants) and green (synonymous variants). Open reading frames affected by missense variants or stop-loss variants are shown in red together with the corresponding protein IDs given as NCBI numbers (WP_xxxxxxxxx.1).

Besides the serological, biochemical and MALDI-TOF MS-based identification tools described above, molecular methods like ribotyping, pulsed-field gel electrophoresis (PFGE), multilocus sequence typing (MLST) or multiple locus variable number of tandem repeats (MLVA) analysis are used for subtyping of *Brucella* spp.^35–37^ and for the differentiation of *B. canis* subclades^38^. As a consequence of the high genetic homology among *Brucella* species^39^, we performed next-generation sequencing (NGS) analyses for a more sophisticated phylogenetic characterization of the *Brucella* isolates in our study. Analyzing the assembled DNA sequencing short reads clearly identified all isolated bacteria as *B. canis* with an average draft genome size of 3,295,525 bp and an average G + C content of 57.27% (Fig. 5 and Supplementary Table S5). Excluding lower quality sequences, the applied short read sequencing technique allowed de novo genome assemblies with 24 to 57 contigs. Repetitive elements like IS711, ISBm2 and ISBm3, IS1953 and IS2020 insertion sequences or rRNA operons hampered the completion of contiguous, circular chromosome sequences, a typical drawback of Illumina and other next-generation sequencing techniques based on short read lengths^40^.

Detailed genome sequence comparisons revealed a high clonality, with only seven SNPs among the *B. canis* isolates (Supplementary Table S6). Apart from isolate I16, which harbored mutations at two different positions, five *B. canis* isolates (I5, I8, I19, I22, I23) differed only by one nucleotide within their genome sequences, which strongly indicates a clonal origin of these bacteria. In four cases, nucleotide variations were identified in *Brucella* isolates from dogs at later sampling dates (Supplementary Tables S1 and S6). Two SNPs, A2198519T in I8 and C642523T in I23, may be the result of post-isolation, lab-acquired mutations or an artefact of the low sequencing coverage (5 ×) for these nucleotide positions. To create an improved consensus genome, sequence reads of four high-quality draft genomes without SNP deviations among the *Brucella* isolates were concatenated by SPAdes de novo assembly. The resulting *B. canis* BfR-SPBR-consensus genome consists of 23 contigs (≥ 500 bp) and has a size of 3,293,143 bp, which is about 19.6 kb shorter than the reference genome sequence of *B. canis* ATCC 23,365 with 3,312,769 bp.

Sequence variants between the *B. canis* consensus genome described here and the *B. canis* ATCC 23365 genome were determined by applying the SNP calling strategies of BioNumerics and Mauve/ParSNP. The BioNumerics read mapping approach revealed 179 positions with high sequence read coverage and five positions with low sequence read coverage SNPs, whereas the analysis with Mauve identified 183 SNPs in the assembly-based approach. Analyses with these two different bioinformatics tools identified 180 SNP positions in common that distinguish the *Brucella* isolates found in dogs of the kennel from the reference strain *Brucella* ATCC 23365 (Fig. 5). Besides various SNPs in non-coding regions of *B. canis* BfR-SPBR-consensus, 57 and 47 SNPs were identified in coding regions of chromosome 1 and chromosome 2, respectively.

**Figure 6:**
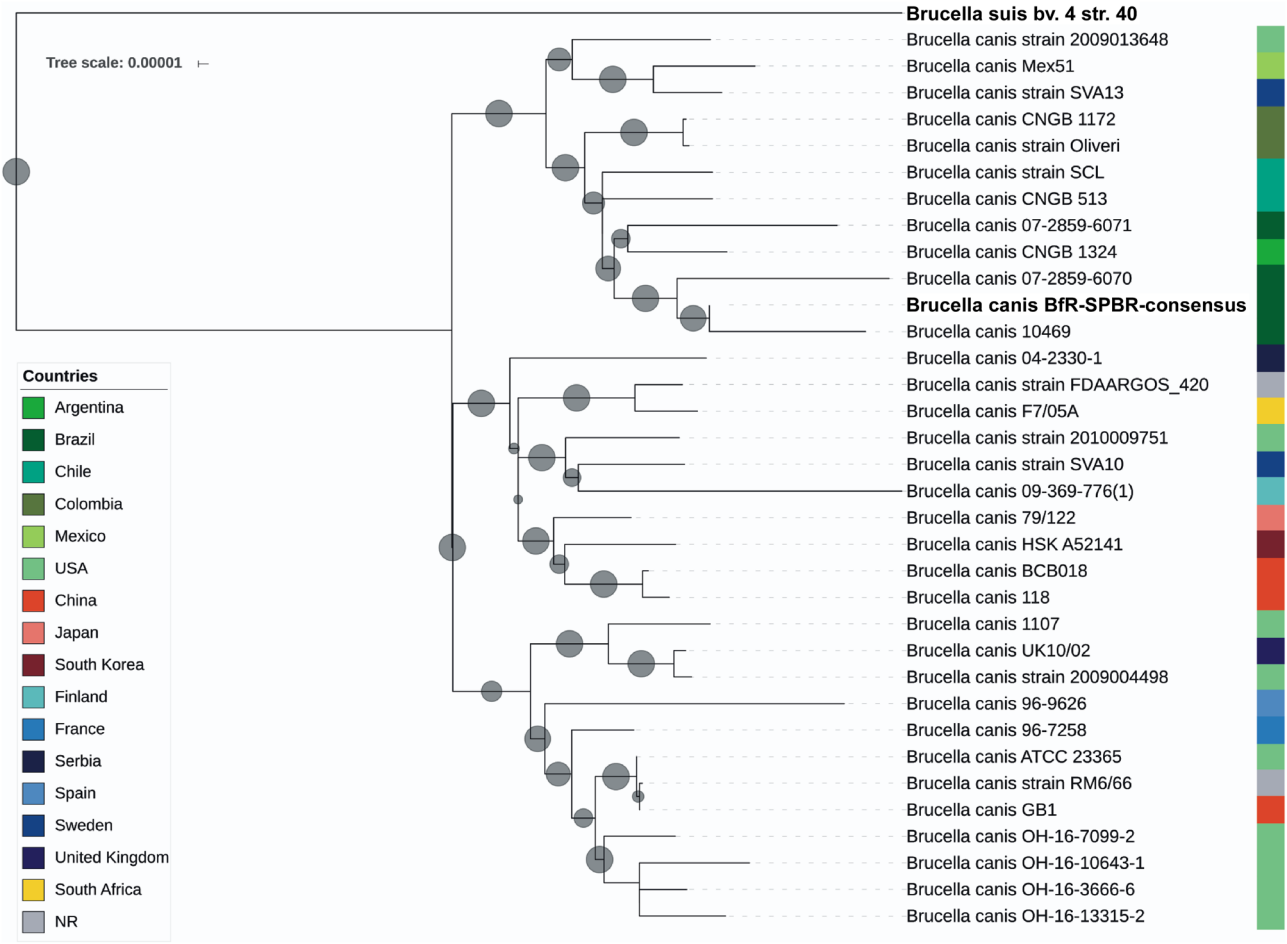
Phylogenetic comparison of the *Brucella canis* BfR-SPBR-consensus strain newly identified in São Paulo with isolates from worldwide outbreaks. The genetic relationship between the outbreak strain *B. canis* BfR-SPBR-consensus and previously sequenced *B. canis* strains was determined by SNP analysis. The genome sequences were analyzed with ParSNP, FastTree2 and iTol. The *B. suis* outgroup strain and the *B. canis* outbreak strain under study are marked in bold. Shown is a neighbor-joining phylogenetic tree with the branch length displaying the relative genetic distance. All bootstrap support values were either below 0.5 or above 0.8.

To illustrate the relationship of *B. canis* BfR-SPBR-consensus with previously sequenced strains, we performed a core genome multi-alignment with ParSNP using all publicly available *B. canis* draft or complete genome sequences and included the *B. suis* bv 4 reference strain 40 as the outgroup (Fig. 6). This analysis clearly placed the *B. canis* BfR-SPBR-consensus in a clade together with other Brazilian *B. canis* isolates but also with strains from neighboring countries. The most closely related strains to *B. canis* BfR-SPBR-consensus were *B. canis* 10469 and *B. canis* 07-2859-6070, also isolated from kennels in São Paulo in 2005 and 1998^41^, respectively, whereas the genome sequences of *B. canis* strains from Chile and Colombia revealed more genetic variations. Interestingly, the human *B. canis* CNGB 1324 isolate from Argentina clustered within the group of Brazilian *B. canis* isolates from dogs (Fig. 6).

**Figure 7:**
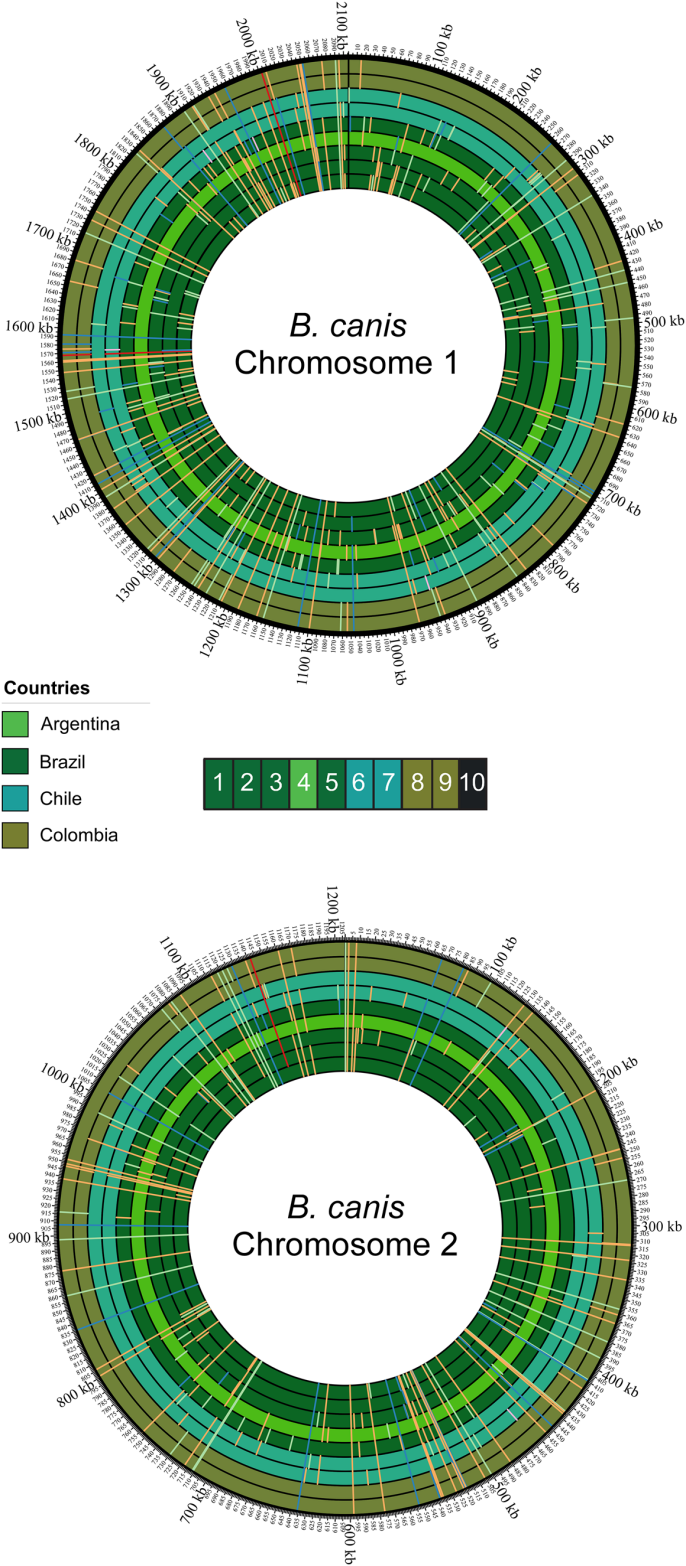
Distribution of single-nucleotide polymorphisms (SNPs) in South American *Brucella canis* isolates. The positions of SNPs in the genomes of South American *B. canis* strains are shown in comparison to the reference strain *B. canis* ATCC 23365. The two chromosomes of the *B. canis* isolates are color-coded and the background colors show the geographical origin for each strain. The strains are presented in the following order from inside to outside: 10469 (1), *B. canis* BfR-SPBR-consensus (2), 07-2859-6070 (3), CNBG 1324 (4), 07-2859-6071 (5), CNGB 513 (6), SCL (7), Oliveri (8), CNGB 1172 (9) and ATCC 23365 (10). SNPs are shown as colored strokes in red (stop-loss variants), in light pink (stop-gain variants), orange (missense variants), blue (upstream or downstream variants) and green (synonymous variants).

Detailed comparison of the SNP distribution in *B. canis* BfR-SPBR-consensus and other South American *B. canis* strains found several conserved missense and synonymous mutations in coding regions, which were absent in *B. canis* ATCC 23365 (Fig. 7). SNPs leading to stop-loss mutations in genes were conserved in most of the South American *B. canis* isolates as well (Table 1). In contrast, SNPs resulting in stop-gain mutations were less frequent and occurred especially in *B. canis* strains from Colombia and Chile (Table 1). The described stop-loss and stop-gain mutations affected genes with physiological functions like nutrient utilization but also potential classical virulence traits such as an autotransporter protein of the type V secretion family, including adhesins, invasins, toxins and proteases, found in various bacterial pathogens^42^.

**Table 1:**
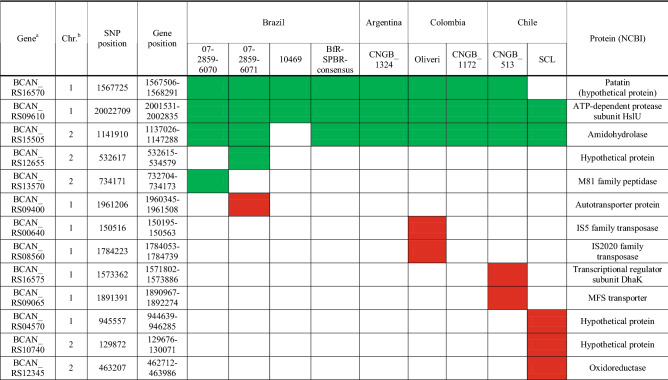
Point mutations of South American *Brucella canis* strains affecting gene functions.

^a^Gene: gene locus of *Brucella canis* ATCC23365; ^b^Chr.: chromosome; green = stop-loss mutation; red = stop-gain mutation.

Summarizing, our genetic analysis clearly reveals the clonality of the *Brucella* isolates from the diseased kennel in São Paulo and suggests a single-strain outbreak. Moreover, we identified SNPs in the South American *B. canis* isolates that may indicate diverse phenotypic properties of representatives within this subclade in spite of their close genetic relationship.

## Discussion

Infections of dogs with *B. canis* and *B. suis* may be asymptomatic or lead to similar clinical manifestations in the infected animals like reproductive failures due to infertility, abortion, stillbirth, orchitis, epididymitis and prostatitis^10,43,44^. In recent years, not only dog-to-dog and pig-to-dog transmissions of *B. canis* and *B. suis,* respectively, have been reported, but both species have also been transmitted from dogs to humans. Hence, canine brucellosis mainly caused by *B. canis* but also by *B. suis* is an emerging zoonosis worldwide^18,26,27,45–48^. Human infection may lead to various unspecific symptoms such as prolonged fever, shivering, chills, night sweats, weight loss, overall weakness or malaise, headache, enlarged lymph nodes, back pain, and arthralgia^10^. Therefore, fast and reliable laboratory methods for the identification of canine *Brucella* species and for epidemiological investigations on single strains are essential to prevent the spread of infection among dogs and transmission to humans.

For this purpose, we examined a potential brucellosis outbreak in a kennel of 17 dogs by comparing the traditional *Brucella* diagnostic tools with the latest proteomics- and genomics-based methods. Fourteen out of 17 animals exhibited clinical manifestations associated with canine brucellosis. One female and two male dogs were asymptomatic, which raised the question of whether these animals were subclinically infected (healthy carriers) or not infected. Subsequent blood cultures allowed us to isolate *Brucella* from two out of the three dogs confirming previous reports that infected dogs do not necessarily develop disease-specific symptoms^28,43^. Therefore, the isolation of bacteria is considered the gold standard method and the most reliable way to confirm canine brucellosis. Moreover, bacterial isolates are required for phenotypic characterization^49,50^ in order to differentiate between the *Brucella* species potentially infecting dogs^8^. By performing the microbiological and biochemical tests commonly used for the classification of *Brucella* spp., we were able to identify all bacteria isolated from the kenneled dogs as *B. canis*. The phenotypic traits of our isolates were consistent with those of the reference strain *B. canis* RM6/66.

In about 40% of the analyzed blood samples, especially from later sampling dates, the isolation of bacteria was unsuccessful although *Brucella* could be recovered from preceding blood samples of respective animals. Obviously, diagnosis by culture may produce false-negative results, particularly in the later stage of infection when bacteremia ceases or becomes intermittent^51,52^. Although we were unable to isolate *Brucella* from the blood of several infected dogs at later sampling dates, we could still detect *Brucella* DNA by genus-specific PCR analysis, which further confirms that these dogs had been infected with *Brucella*. Our results verify previous observations that blood culture may be insufficient to diagnose canine brucellosis, especially since attempts to recover *B. suis* bv 1 from blood and urine had failed while bacteria could be isolated from the semen of dogs with clinical manifestations of brucellosis^53^.

Because of the drawbacks of blood culture for diagnostic purposes, serodiagnosis is commonly used to detect *Brucella* infections in animals. Serological approaches take advantage of seroconversion in infected hosts and detect host antibodies that react with *Brucella* antigens. These anti-*Brucella* antibodies can still be detected several years after the acute stage of infection^28,43,54^. Our serological screening of the 17 dogs using ICT, ELISA and RSAT suggested that all animals were infected with *Brucella*, even the two dogs with negative blood culture results at any sampling date. However, the results of the three serological tests were inconclusive in various cases, since some serum samples with positive ELISA results showed negative results with ICT or RSAT. Moreover, we observed negative serological test results in samples from which *Brucella* had been isolated. Hence, our analysis confirmed previous studies on the serological diagnosis of canine brucellosis demonstrating that although the detection of antibodies against rough *Brucella* species is widely used, misdiagnosis due to sensitivity and specificity failures may occur when these tests are applied^43,54–58^. False-negative results can be a consequence of testing during the initial phase of infection prior to seroconversion, or of low levels of circulating antibodies in chronically infected dogs, after bacteremia has ceased^51,59^. Serological titers may also wax and wane during bacteremia leading to false-negative results^28^. False-positive results, for instance, may occur as a consequence of cross-reactivity of anti-*Brucella* antibodies directed against other pathogens. For *B. canis* harboring a rough LPS, cross-reactivity with *Streptococcus*, *Staphylococcus*, *Bordetella*, and *Pseudomonas* has been described, whereas the food- and waterborne pathogens *Escherichia coli* O157:H7, *Yersinia enterocolitica* O:9, *Salmonella* Typhimurium (group N; O:30), and *Vibrio cholerae* O1 induce immune responses that generate antibodies reacting against the smooth LPS of *B. suis*^43,60–62^*.*

The species identification of our *Brucella* strains as *B. canis* with traditional microbiological, serological, biochemical and phage typing methods required several working days. Therefore, one focus of our study was to compare MALDI-TOF MS, as a more reliable and faster identification and differentiation tool for *Brucella* spp., with the classical phenotyping tests. Previous studies have shown that the application of MALDI-TOF MS with the currently available public libraries is a highly sensitive and specific technique for genus identification^33,63–65^. However, due to the close genetic relationship among *Brucella* spp., MALDI-TOF MS analyses has not yet allowed for a robust and unambiguous discrimination of *Brucella* species or their biovars. With our present work we significantly improved the current MALDI-TOF MS-based diagnostics of canine *Brucella* species by determining new biomarkers that enabled us not only to distinguish between *B. canis* and *B. suis* bv 1 but also between *B. canis* and the phylogenetically much closer *B. suis* bv 4. *Brucella canis* has previously been characterized by MALDI-TOF MS from blood cultures of infected dogs^63^, but these isolates could only be correctly identified at species level by adding customized main spectra of *B. canis* to the Bruker SR library containing *B. melitensis* as the sole representative of the genus *Brucella*. The obtained Bruker Biotyper identification scores consequently supported identification of *B. canis* with high probability, since the lack of complementary main spectra from other *Brucella* spp. constrains identification results to the species available in the used library. Purvis et al.^63^ did not evaluate the discriminatory power of MALDI-TOF MS analysis for *B. canis* identification against an expanded database containing reference spectra from closely related species, which, in our opinion, is a prerequisite for the reliable identification of *B. canis* and discrimination from *B. suis*. Our here presented weighted pattern matching approach overcomes this shortcoming, and its ability to identify and distinguish *B. suis* bv 1 from *B. suis* bv 4 and *B. canis* provides a valuable benefit for the diagnosis of canine brucellosis. The discriminatory power of MALDI-TOF MS can also be of public health importance because the transmission of *B. suis* has been reported not only from feral pigs and hares to dogs but also from feral pigs to domestic pigs and to humans^18,27,66,67^. The implementation of our optimized MALDI-TOF MS identification approach for *B. canis* and *B. suis* will speed up the specific diagnosis of canine brucellosis as described for the new diagnostic routines with other pathogens^68^.

When we complemented our MALDI-TOF MS analysis with whole genome sequencing data of *Brucella* isolates from the different dogs of the kennel, we were able to determine that the diverse clinical presentations of the animals were indeed the result of a *B. canis* single-strain outbreak. A detailed SNP comparison of the consensus sequence derived from our *B. canis* isolates with published *B. canis* genome sequences clustered the outbreak clone with various strains from South America comprising a clade that was clearly distinguishable from *B. canis* isolates from other parts of the world^41^. The genetic proximity of the Brazilian and Argentinian *B. canis* strains indicates that the infection circulates in kennels by cross-border trade of dogs, which may finally lead to a high risk for public health due to the close contact of humans with dogs and the lack of surveillance for canine brucellosis. The close genetic relationship of certain *B. canis* strains isolated from dogs and humans hints to a yet underestimated zoonotic transmission of this pathogen. Such transmission was recently documented with the infection of a 3-year old child by the same *B. canis* strain found in the blood of the child’s puppy^46^.

The close phylogenetic relationship of the South American *B. canis* strains based on the SNPtree analysis suggested that they represent a homogenous group with similar properties. However, our detailed and genome-wide analysis of single nucleotide mutations revealed that various SNPs affect coding regions either resulting in gene inactivation or activation of pseudogenes. Consequently, this genetic microdiversity might lead to more pronounced functional differences than their genetic homology may imply. Future studies have to address the extent to which such phenotypic differences exist and how they might affect the virulence of specific *B. canis* strains.

Our study supports previous notions^7^ that *B. canis* is prevalent in Brazilian breeding kennels and can be readily spread. These cases highlight the need to assess potential public health risks associated with the trade of kennel dogs or the handling of hunting dogs. In this context, new surveillance and control measures are indispensable to protect animal and human health. The rapid identification of the pathogen involved in kennel outbreaks is an important step to prevent the spreading of canine brucellosis. When the infection is confirmed in a dog population, the detailed characterization of the *Brucella* strains is required to trace back the infection. Since the members of the genus *Brucella* show high DNA sequence identity and clonal evolution, high-resolution typing methods are necessary to perform epidemiological analyses. Our study illustrates that proteomics and genomics approaches are a necessary complement to the traditional tests used in *Brucella* diagnostics because these novel techniques may speed up the reliable diagnosis of canine brucellosis.

## Methods

### Serodiagnosis of canine brucellosis

Blood sampling from dogs was performed according to a protocol approved by the Ethics Committee of the Faculty of Veterinary Medicine and Animal Science at the University of São Paulo, Brazil, under protocol CEUA 3113091015/2015, and in accordance with the relevant guidelines and regulations of the National Council for Control of Animal Experimentation (CONCEA). All blood samples were collected after mutual consensus between the owner of the kennel and the veterinarians responsible. Blood samples (~ 5 ml) were aseptically taken by cephalic or jugular venipuncture from dogs in a kennel located in São Paulo, Brazil**.** Half of the collected blood volume was mixed with sodium citrate as an anticoagulant. The remaining blood clotted at room temperature, was centrifuged and serum was stored at − 20 °C until use in serological tests. To test for anti-*B. canis* antibodies, we performed immunochromatographic tests (ICT; Rapid Canine *Brucella* Ab Test Kit, Bionote, Hwaseong, South Korea), ELISA (Novateinbio Kit, Cambridge, MA, USA) and rapid slide agglutination tests (RSAT; Canine Brucellosis Antibody Test Kit, D-TEC CB, Kansas City, MO, USA) with or without 2-mercaptoethanol (2ME) according to manufacturers’ instructions. The 2ME-RSAT was only conducted on serum samples tested positive by RSAT.

### Isolation of *B. canis* from blood culture and phenotypic characterization

Full blood samples containing sodium citrate were used for culture as previously described^69^. Briefly, enrichment culture was performed in tryptose phosphate broth (Difco) including 5% fetal calf serum (FCS; Cultilab) at 37 °C for 30 days, followed by subcultures every four days on solid tryptose agar, also supplemented with 5% FCS. The isolated bacteria were primarily screened to confirm *Brucella* spp. colonies using the genus-specific PCRs targeting IS711^70^ and *bcsp*31^71^. All *Brucella* isolates collected on the first and second sampling date (Supplementary Table S1) were further characterized using morphological, biochemical and metabolic tests, such as Gram, Stamp and crystal violet staining, CO_2_ requirement, H_2_S production, oxidase and catalase activity, urea hydrolysis, agglutination with monospecific sera (anti-A, anti-M, and anti-R), dye sensitivity (basic fuchsin and thionine), bacterial motility (triphenyl tetrazolium chloride solid agar), and phage lysis (F1, F25, Tb, BK2, Iz, Wb, R/C) in order to identify and sub-differentiate *Brucella* spp.^30^.

### Polymerase chain reaction (PCR) to detect *Brucella* DNA in canine blood samples

The whole blood samples were also used for direct detection of *Brucella* DNA by IS711 PCR when bacterial isolation was not successful. Briefly, the extraction of the bacterial DNA was based on mechanical and enzymatic pre-lysis of leukocytes, using zirconia/silica beads and lysozyme, respectively, followed by overnight enzymatic lysis using proteinase K and sodium dodecyl sulphate (SDS). The DNA was finally purified by phenol/chloroform and alcohol precipitation as described previously^71,72^. DNA gel electrophoresis images of the PCR products were edited using Adobe Photoshop CS5 (Adobe Systems, San Jose, CA, USA).

### Matrix-assisted laser desorption/ionization-time of flight mass spectrometry (MALDI-TOF MS) to sub-differentiate *Brucella* species

*Brucella* isolates were primarily inactivated in 75% ethanol for at least 90 min and stored at − 20 °C until use. Samples were prepared for mass spectrometry by ethanol-formic acid extraction according to manufacturer’s instructions before being spotted on a 96-spot steel plate target and covered with alpha-cyano-4-hydroxy-cinnamic acid (HCCA) matrix solution (Bruker Daltonics). Mass spectra were acquired using a Bruker Microflex LT MALDI-TOF MS system (Bruker Daltonics). Spectra were initially analyzed using the Bruker Biotyper 3.1 software with MSP library version MBT 7311 (7311 entries) and the Security-Relevant (SR) Database (104 entries). None of the commercial libraries contained reference spectra of *B. canis*. Therefore, we complemented the database with in-house mass spectra of various *B. canis* and *B. suis* strains (obtained from Karger et al. (2013)^32^ and this study).

For each sample, a total of 24 spectra were measured, analyzed and visualized with the commercial Biotyper software as well as the open source statistical computing environment R v3.6.3^73^ using the packages MALDIquant v1.19.3^74^ and Tidyverse v1.3.0^75^.

Bruker Biotyper identification scores for each sample were calculated from a main spectrum (MSP) composed of the 20 best quality technical replicate spectra following manufacturer’s instructions. Identification scores were interpreted accordingly, with scores ≥ 2.3 indicating highly probable species identification, scores between 2 and 2.3 indicating secure genus identification and probable species identification, scores between 1.7 and < 2 indicating probable genus identification and scores < 1.7 indicating no reliable identification.

Spectra processing with MALDIquant was performed similar to MSP creation by calculating an averaged spectrum of all technical replicate spectra of a sample. Averaged group spectra represent the mean of all available *Brucella* field isolates and reference strains for the respective species or biovar.

### Next generation sequencing (NGS) of the *Brucella canis* isolates

#### Sample preparation

*Brucella canis* was grown on *Brucella* Agar and the DNA of the bacteria was extracted using the PureLink Genomic DNA extraction kit (Invitrogen) according to the manufacturer’s protocol. The libraries for whole-genome sequencing were prepared with a Nextera XT sample preparation kit. An Illumina MiSeq platform with a 2 × 300 cycle, paired-end configuration was used for sequencing.

#### Bioinformatics analysis

Sequence read quality was analyzed with FastQC v0.11.5 (Babraham Bioinformatics). The sequence reads and publicly available read files from the Sequencing Read Archive (https://www.ncbi.nlm.nih.gov/sra/) were assembled with SPAdes v3.10.0 (BayesHammer read error correction) and the options –*careful* (post-processing mismatch corrector with BWA) and *–cov-cutoff auto*^76^. Assembly quality was analyzed using Quast v4.5^77^ by comparison to the type strain *B. canis* ATCC 23,365 genome (GenBank file GCF_000018525.1, accession no. NC_010103.1 for chromosome 1 and accession no. NC_010104.1 for chromosome 2 with a genome size of 3,312,769 bp in total). Sequence reads were mapped with BowTie2 v2.3.0^78^ using default parameters against the complete genome of *B. canis* ATCC 23365 or the consensus genome of the *B. canis* outbreak strain under study, assembled from concatenated read files of high quality samples (I9, I13, I14 and I15), hereafter named “*Brucella canis* BfR-SPBR-consensus”.

Mapping statistics were analyzed with QualiMap v2.2.1^79^. To determine the average nucleotide identity (ANI), Gegenees v2.2.1^80^ was applied with a fragment size of 200. Insertion sequences (IS) were identified by online BLASTn analysis against the IS database of the ISfinder website^81^. Inspection of the De Bruijn assembly graph was done with Bandage v1.0^82^. For molecular genotyping, in silico multi-locus variable number of tandem repeat analysis (MLVA-16) was calculated with a Python script^83^ and in silico multi-locus sequence typing (MLST-21) was calculated with the *mlst* program by Torsten Seemann (https://github.com/tseemann/mlst).

Genetic relatedness between the Brazilian isolates and publicly available sequences was determined with ParSNP v1.0^84^. To this end, all available sequences including *B. canis* genomes in the PATRIC (www.patricbrc.org) or NCBI (https://www.ncbi.nlm.nih.gov/genome/microbes/) database as well as draft genomes assembled from read files retrieved from the Sequencing Read Archive (https://www.ncbi.nlm.nih.gov/sra/) and the *B. canis* BfR-SPBR-consensus genome were collected. Maximum-Likelihood trees were calculated by FastTree2^85^. The origin of strains was extracted from genome metadata and integrated into the phylogeny with the EMBL interactive tree of life, iTOL v4^86^, for visualization.

#### Single nucleotide polymorphism (SNP) analysis

Whole genome single nucleotide polymorphism (wgSNP) analysis was conducted using two different strategies, a read-based approach with BioNumerics v7.6 (Applied Maths, Sint-Martens-Latem, Belgium) and an assembly-based approach with Mauve v2015-02-13^87^.

For the BioNumerics procedure, reads were trimmed in a consecutive manner: first with the configuration “structural trimming (remove stretches: homopolymer ≤ 20; polyA > 90%; polyGC < 10% & > 90%), tail quality trimming (tail q ≥ 18; windowed average q ≥ 15; rolling average q ≥ 15), length trimming (exclude < 100 bp & > 250 bp)”, and second with the configuration “overall quality trimming (exclude minimum q window = 5; average q < 20; replace with N for q < 15)”, and were mapped to either the complete genome of *B. canis* ATCC 23365 or the *B. canis* BfR-SPBR-consensus genome.

The resulting SNP matrix was filtered for positions with a minimum coverage of five reads in total, with at least one SNP on the forward and the reverse strand. Due to the filter limitations of BioNumerics, SNP positions were extracted and manually curated in Microsoft Excel 2013 to differentiate between false base calls in the de novo assembly and SNPs at positions present in all 24 sequences or at positions with ambiguous base calls or insufficient coverage (N). Based on the final SNP matrix the pairwise distance between the isolates was calculated and clustering of isolates was performed using the Neighbor Joining algorithm in BioNumerics.

A comparison of *B. canis* BfR-SPBR-consensus against reference sequences including variant calling was performed in Mauve v2015-02-13. An identical SNP list was extracted using ParSNP v1.0 and hence could be used for SNP annotation by SnpEff^88^ with the RefSeq annotation of reference GCF_000018525.1.

#### Nucleotide sequence accession numbers

The lllumina MiSeq raw sequence reads used to generate the draft *B. canis* BfR-SPBR-consensus genome sequence have been deposited in the database of the European Nucleotide Archive (ENA) under the accession numbers ERX4130724, ERX4130725, ERX4130726 and ERX4130727 within the BioProject ERP121782.

## Supplementary information

Supplementary Information
