# Supplementary material for: MALDI-TOF MS and genomic analysis can make the difference in the clarification of canine brucellosis outbreaks
Source: Sci Rep. 2020 Nov 6;10:19246. doi: 10.1038/s41598-020-75960-3 (PMC7648634; doi:10.1038/s41598-020-75960-3)
Supplement: Supplementary file 1 — Supplementary Information [file 41598_2020_75960_MOESM1_ESM.docx]

# Supplementary Information for:

**MALDI-TOF MS and genomic analysis can make the difference in the clarification of canine brucellosis outbreaks**

David Attuy Vey da Silva^1,2#^, Holger Brendebach^1#^, Josephine Grützke^1^, Ralf Dieckmann^1^,
Rodrigo Martins Soares^2^, Julia Teresa Ribeiro de Lima^2^, Lara Borges Keid^3^,
Dirk Hofreuter^1*^ and Sascha Al Dahouk^1,4^

^1^Department of Biological Safety, German Federal Institute for Risk Assessment, Berlin, Germany

^2^Department of Preventive Veterinary Medicine and Animal Health, Faculty of Veterinary Medicine and Animal Science, University of São Paulo, São Paulo, Brazil

^3^Department of Veterinary Medicine, Faculty of Animal Science and Food Engineering, University of São Paulo, Pirassununga, Brazil

^4^Department of Internal Medicine, RWTH Aachen University Hospital, Aachen, Germany

^#^ These authors contributed equally to this work.

* Correspondence: Dirk.Hofreuter@bfr.bund.de

# Supplementary Figures


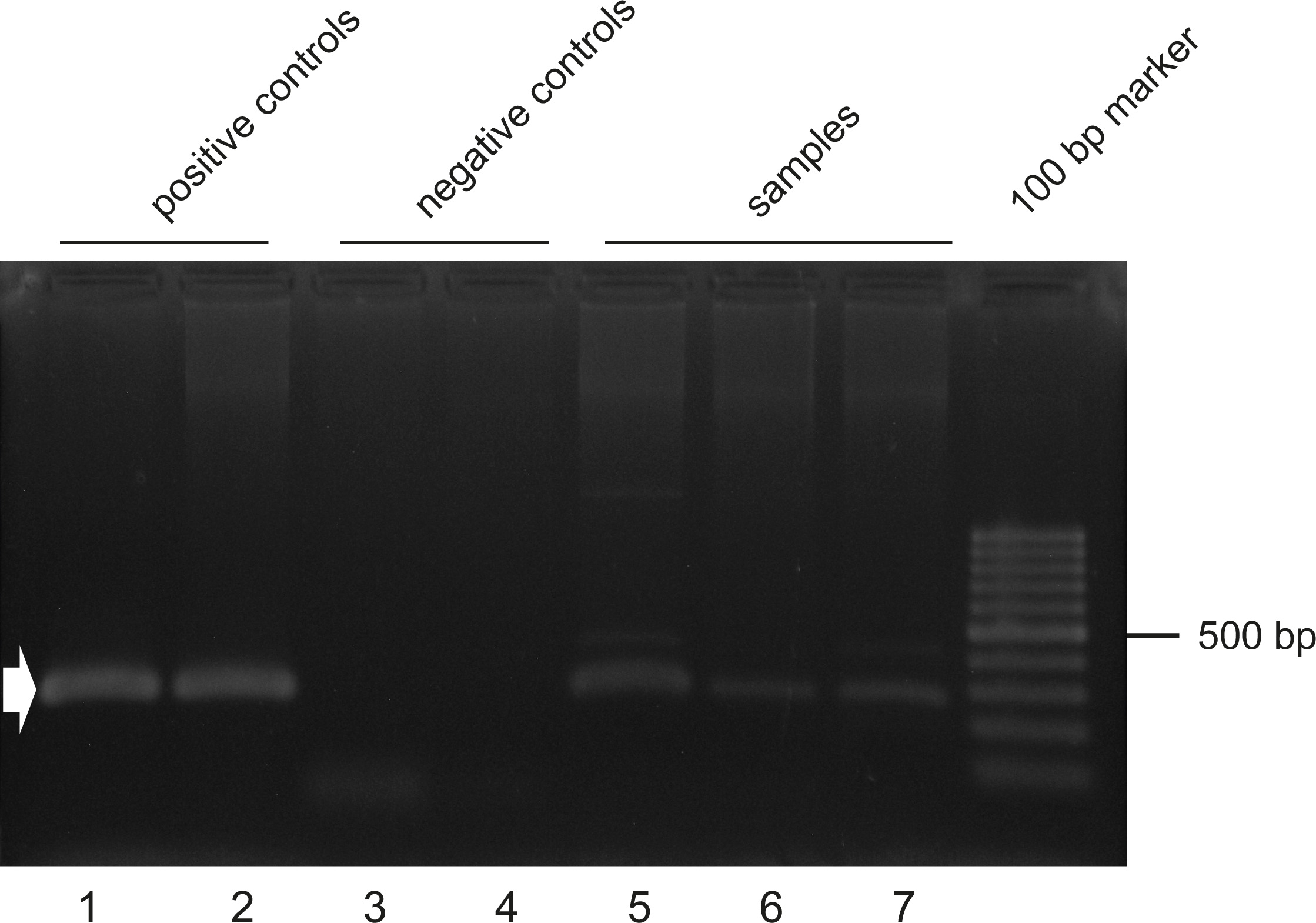


**Supplementary Figure S1.** Results of the IS711 PCR targeting a 262 bp fragment specific for the genus *Brucella* visualized by gel electrophoresis. Purified genomic DNA of the reference strain *B. canis* RM6/66 (1) and from a *B. canis*-positive blood culture (2) were used as positive controls. The negative controls were distilled water as PCR amplification (3) and TE buffer as extraction control (4). PCRs with DNA preparations of blood samples taken from three dogs with *Brucella*-negative blood culture (D07 on 2 Aug 2014, D06 on 8 Nov 2014, and D17 on 8 Nov 2014; see Supplementary Table S1) resulted in IS711-specific amplification products (5, 6 and 7, respectively).

**
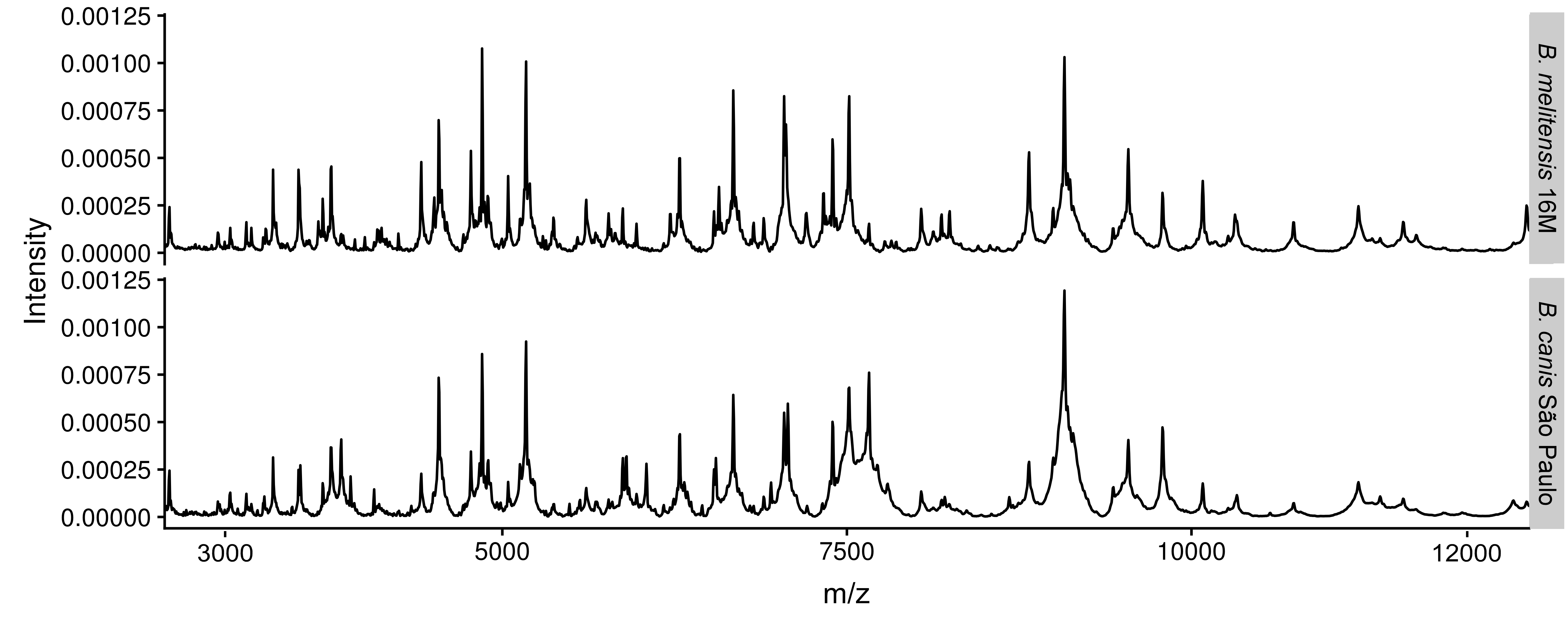
**

**Supplementary Figure S2. MALDI-TOF MS profile of *B. canis* and *B. melitensis*.**


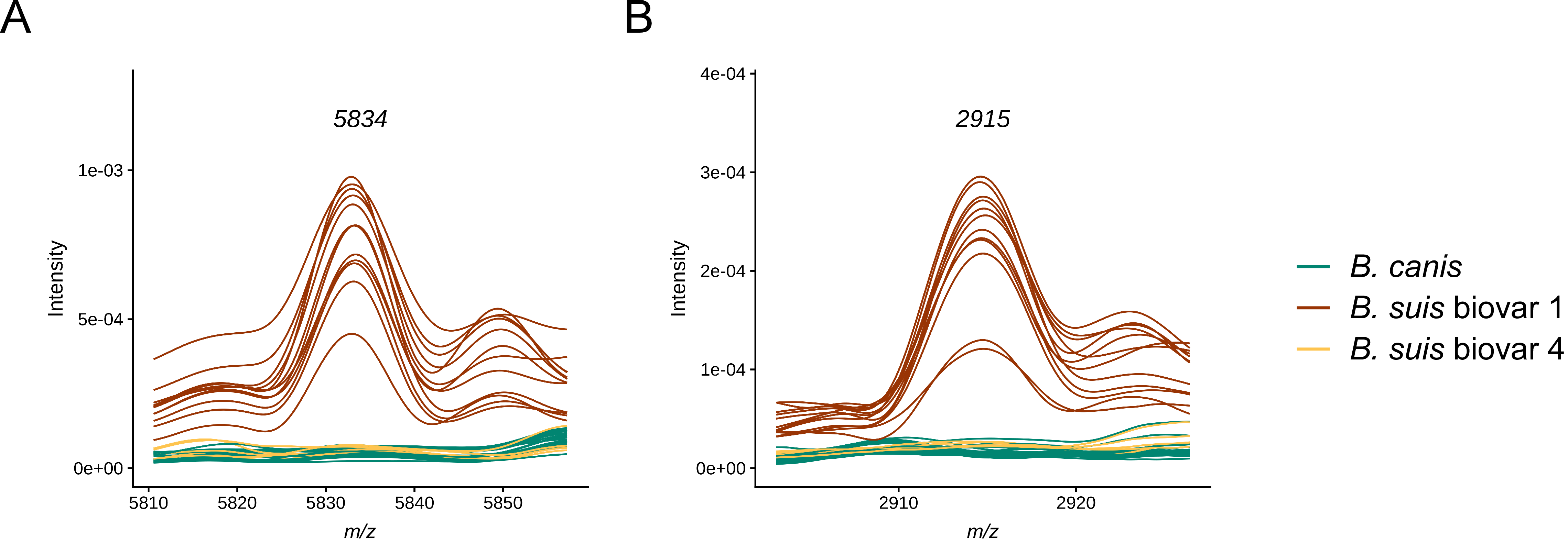


**Supplementary Figure S3. Previously published biomarker for the differentiation of *B. canis*/*B. suis* bv 4 and *B. suis* bv 1. (A) Single charged ion at m/z 5834 as published in Karger *et al.*^[1]^. (B) Double charged ion of the same molecule (this study).**

1 Karger, A. *et al*. Interlaboratory comparison of intact-cell matrix-assisted laser desorption ionization-time of flight mass spectrometry results for identification and differentiation of *Brucella* spp. *J. Clin. Microbiol.* **51**, 3123-3126, 10.1128/jcm.01720-13 (2013).


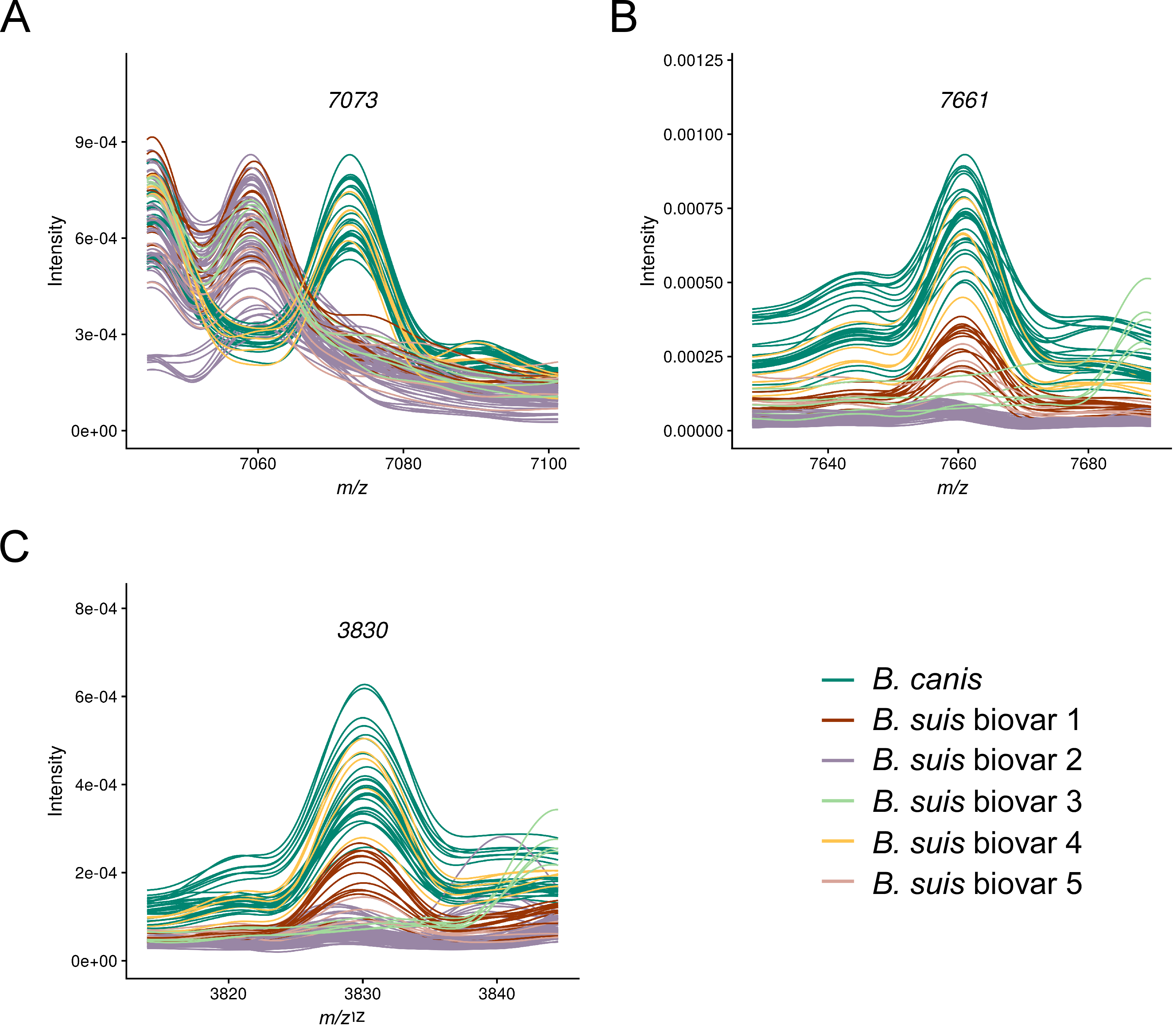


**Supplementary Figure S4.** New biomarkers supporting the differentiation between *B. canis* and *B. suis.* (A) A unique biomarker for the group *B. canis* and *B. suis* bv 4 with the mass peak at m/z 7073 facilitates their distinction from other biovars of *B. suis*; (B, C) *B. canis* and *B. suis* bv 4 may be discriminated from *B. suis* bv 1 by their higher intensity mass peaks at m/z 7661 (single charged) and m/z 3830 (double charged). Additionally, these biomarker peaks enable a clear differentiation between *B. canis* and *B. suis* bv 2 as well as *B. suis* bv 3.

# Supplementary Tables

**Supplementary Table S1. Identification code of the samples and isolates drawn from 17 dogs of a kennel in São Paulo, Brazil.**

| Dog | 22^nd^ March 2014 | | 2^nd^ August 2014 | | 8^th^ November 2014 | | |
| --- | --- | --- | --- | --- | --- | --- | --- |
|  | Serum sample | *Brucella canis*  isolate | Serum sample | *Brucella canis*  isolate | Serum sample | *Brucella canis*  isolate | |
| D07 | S07 | I2 | S78 | n.g. | S112 | n.g. |  |
| D12 | S12 | I5 | S86 | n.g. | S120 | n.g. |  |
| D13 | S13 | I6 | S80 | n.g. | S114 | n.g. |  |
| D20 | S20 | I12 | S87 | n.g. | S121 | n.g. |  |
| D21 | S21 | I13 | S88 | n.g. | S122 | n.g. |  |
| D06 | S06 | I1 | S73 | I16 | S107 | n.g. |  |
| D14 | S14 | I7 | S81 | I21 | S115 | n.g. |  |
| D17 | S17 | I9 | S84 | I24 | S118 | n.g. |  |
| D19 | S19 | I11 | S72 | I15 | S106 | n.g. |  |
| D22 | S22 | I14 | S79 | I20 | S113 | n.g. |  |
| D09 | S09 | I3 | S76 | I18 | S110 | I25 (n.i.) |  |
| D10 | S10 | I4 | S75 | I17 | S109 | I26 (n.i.) |  |
| D16 | S16 | I8 | S82 | I22 | S116 | I27 (n.i.) |  |
| D18 | S18 | I10 | S83 | I23 | S117 | I28 (n.i.) |  |
| D15 | S15 | n.g. | S77 | I19 | S111 | I29 (n.i.) |  |
| D08 | S08 | n.g. | S85 | n.g. | S119 | n.g. |  |
| D11 | S11 | n.g. | S74 | n.g. | S108 | n.g. |  |

n.g.: no growth; n.i.: not investigated

**Supplementary Table S2. Phenotypic characterization of *Brucella* isolates from the dogs of the kennel under study.**


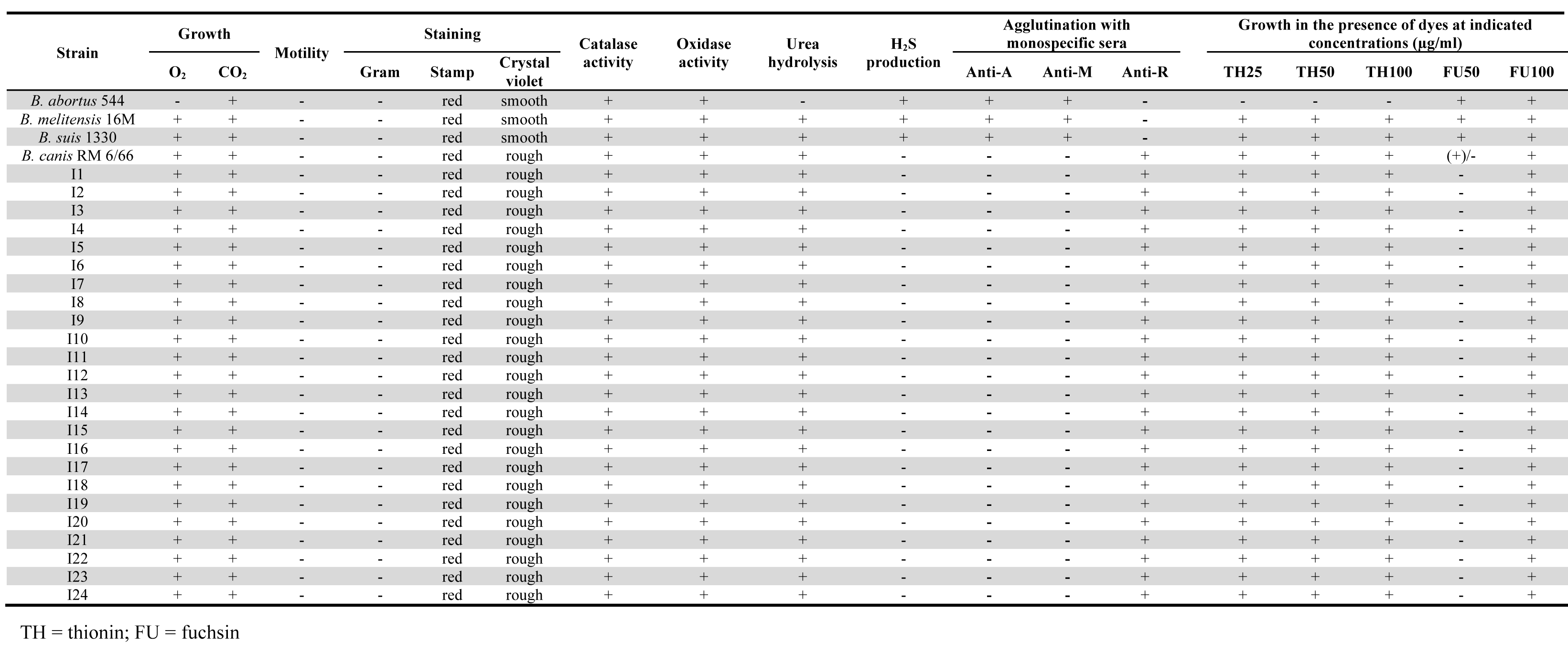


**Supplementary Table S3. Phage typing of *Brucella abortus*, *B. melitensis*, and *B. canis* reference strains.**

| Reference | Strain | Phages | | | | | | | |
| --- | --- | --- | --- | --- | --- | --- | --- | --- | --- |
|  |  | Tb | Wb | BK2 | F21 | F25 | Iz | Fi | R/C |
| This study | *B. abortus* 544 |  |  |  |  |  |  |  |  |
|  | *B. melitensis* 16M |  | **v** |  |  |  |  |  |  |
|  | *B. canis* RM 6/66 |  |  |  |  |  |  |  |  |
|  |  |  |  |  |  |  |  |  |  |
| Hammerl *et al.* **^[^**^1^**^]^** | *B. abortus* 544 |  |  |  | nc | nc |  |  |  |
|  | *B. melitensis* 16M |  |  |  | nc | nc |  |  |  |
|  | *B. canis* RM 6/66 |  |  |  | nc | nc |  |  |  |
|  |  |  |  |  |  |  |  |  |  |
| Kang *et al.* **^[^**^2^**^]^** | *B. abortus* 544 |  |  |  | nc | nc | nc | nc | nc |
|  | *B. melitensis* 16M |  |  |  | nc | nc | nc | nc | nc |
|  |  |  |  |  |  |  |  |  |  |
|  | *B. abortus* |  |  |  | nc | nc |  |  |  |
| Corbel *et al.* **^[^**^3^**^]^** | *B. melitensis* |  | **v** |  | nc | nc |  |  |  |
|  | *B. canis* |  |  |  | nc | nc |  |  |  |

Dark gray: lysis; light gray: no lysis; v: variable; nc: not conducted

1 Hammerl, J. A. *et al.* Genetic diversity of *Brucella* reference and non-reference phages and its impact on *Brucella*-typing. *Front* *Microbiol* **8**, 408-408, doi:10.3389/fmicb.2017.00408 (2017).

2 Kang, Y.-X. *et al.* Typing discrepancy between phenotypic and molecular characterization revealing an emerging biovar 9 variant of smooth phage-resistant *B. abortus* strain 8416 in China. *Front Microbiol* **6**, 1375-1375, doi:10.3389/fmicb.2015.01375 (2015).

3 Corbel, M. J. *Brucella* phages: Advances in the development of a reliable phage typing system for growth and non-smooth *Brucella* isolates. *Ann Inst Pasteur Microbiol* **138**, 70-75, doi:https://doi.org/10.1016/0769-2609(87)90056-1 (1987).

**Supplementary Table S4. List of strains included in the MALDI-TOF MS analysis.**


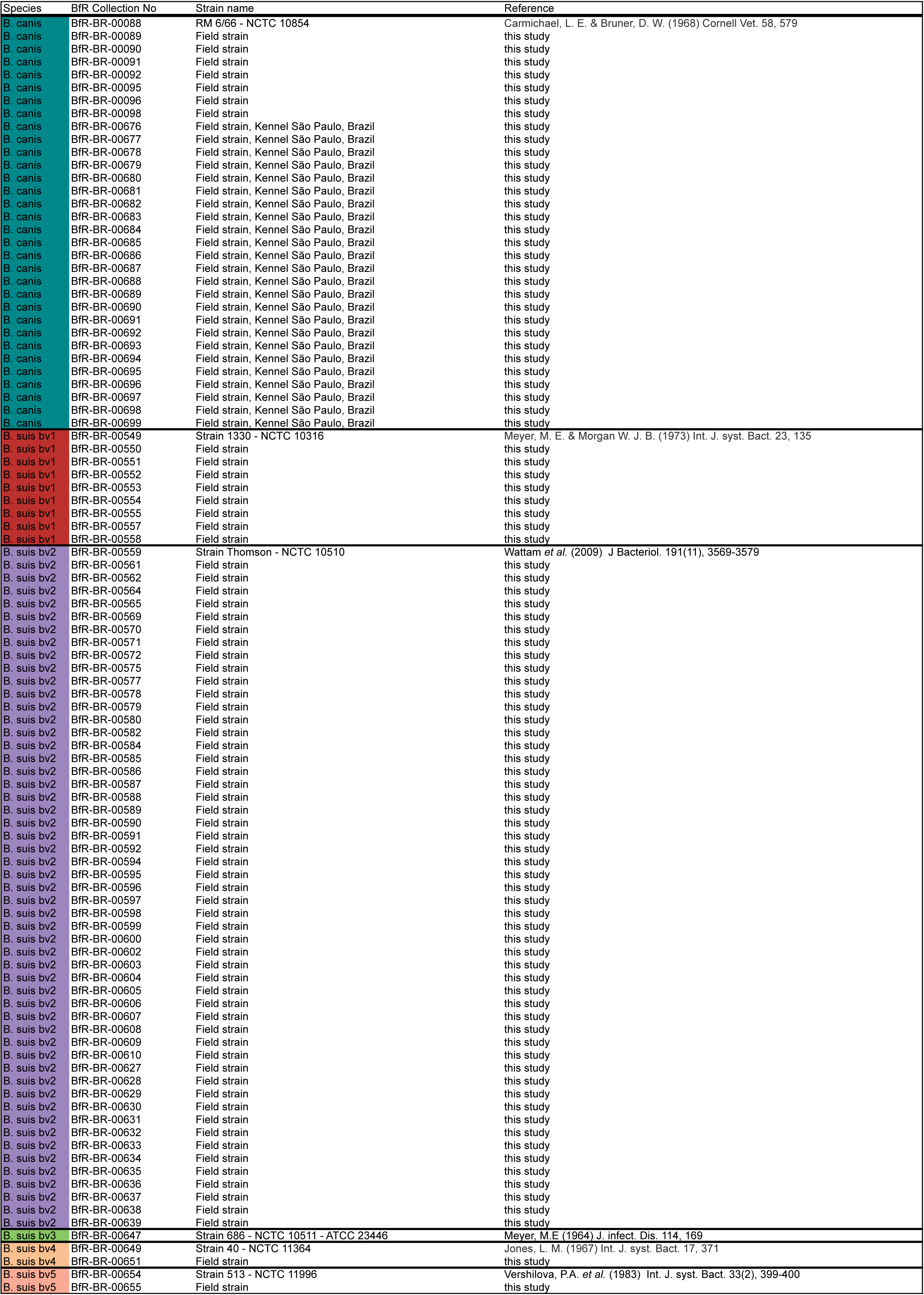
**Supplementary Table S5. Next-generation sequencing and assembly quality report.**


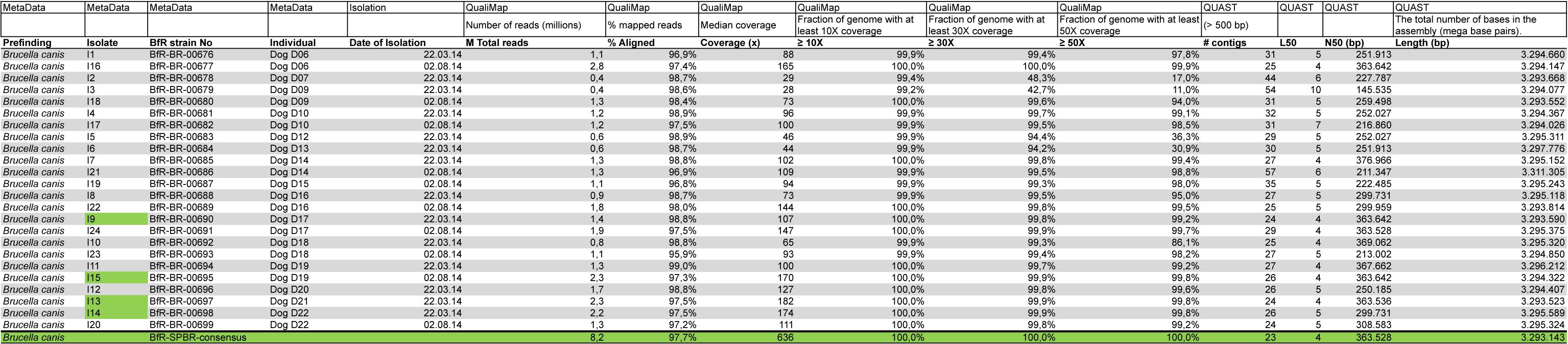


**Genome sequences of the four *B. canis* isolates that were used for generating the *B. canis* BfR-SPBR-consensus sequence are marked in green**

**Supplementary Table S6. SNP analysis among the 24 *Brucella canis* isolates belonging to our outbreak investigation.**

| Dog | Isolate | Position in ATCC 23365 genome | | | | | | |
| --- | --- | --- | --- | --- | --- | --- | --- | --- |
|  |  | 642523 | 997022 | 1104956 | 1402095 | 1463341 | 2196420 | 2198519 |
| D07 | I2 | C | G | G | C | G | C | A |
| D12 | I5 | C | G | G | T | G | C | A |
| D13 | I6 | C | G | G | C | G | C | A |
| D20 | I12 | C | G | G | C | G | C | A |
| D21 | I13 | C | G | G | C | G | C | A |
| D06 | I1 | C | N | G | C | G | C | A |
|  | I16 | C | G | G | C | A | T | A |
| D14 | I7 | C | G | G | C | G | C | A |
|  | I21 | C | G | G | C | G | C | N |
| D17 | I9 | C | G | G | C | G | C | A |
|  | I24 | C | G | G | C | G | C | A |
| D19 | I11 | C | G | G | C | G | C | A |
|  | I15 | C | G | G | C | G | C | A |
| D22 | I14 | C | G | G | C | G | C | A |
|  | I20 | C | G | G | C | G | C | N |
| D09 | I3 | C | G | G | C | G | C | A |
|  | I18 | C | G | G | C | G | C | A |
| D10 | I4 | C | G | G | C | G | C | A |
|  | I17 | C | G | G | C | G | C | N |
| D16 | I8 | C | G | G | C | G | C | T |
|  | I22 | C | G | A | C | G | C | A |
| D18 | I10 | C | G | G | C | G | C | A |
|  | I23 | T | G | G | C | G | C | A |
| D15 | I19 | C | A | G | C | G | N | A |
| RC | | 5 | 13 | 32 | 20 | 80 | 52 | 5 |

C – Cytosine; T – Thymine; G – Guanine; A – Adenosine; RC – Read coverage in the divergent isolate; N – Ambiguous nucleotides
